# Artificial intelligence prediction of nonenhancing brain tumor malignancy based on *in vivo* confocal laser endomicroscopic imaging

**DOI:** 10.3389/fsurg.2025.1655374

**Published:** 2026-01-05

**Authors:** Jiuxu Chen, Yuan Xu, Irakliy Abramov, Carlos E. Calderón-Valero, Thomas J. On, Jennifer M. Eschbacher, Baoxin Li, Mark C. Preul

**Affiliations:** 1 School of Computing and Augmented Intelligence, Arizona State University, Tempe, AZ, United States; 2 The Loyal and Edith Davis Neurosurgical Research Laboratory, Barrow Neurological Institute, St. Joseph’s Hospital and Medical Center, Phoenix, AZ, United States; 3 Department of Neuropathology, Barrow Neurological Institute, St. Joseph’s Hospital and Medical Center, Phoenix, AZ, United States

**Keywords:** artificial intelligence, deep learning, computer vision, confocal laser endomicroscopy, nonenhancing brain tumor, low-grade glioma, high-grade glioma

## Abstract

**Background:**

Although nonenhancing tumors are often thought to be lower grade, malignant regions can be missed on conventional magnetic resonance imaging. Fluorescein-based confocal laser endomicroscopy (CLE) enables real-time, cellular-resolution imaging of brain tissue during tumor resection. It is particularly valuable for evaluating nonenhancing brain tumors. However, CLE interpretation remains subjective. Although CLE has high sensitivity, it is less specific than standard histology. Existing artificial intelligence (AI) models process CLE images as independent frames, neglecting the temporal context that human experts use during interpretation.

**Methods:**

A novel sequence-based deep learning model was developed to classify tumor grade on the basis of CLE image sequences, mimicking the visual reasoning process of expert neuropathologists. CLE images were collected from 16 patients with nonenhancing brain tumors. Each sequence was labeled as high grade or low grade based on neuropathologist interpretation, blinded to final histopathology findings. Visual features were extracted using pretrained backbones (vision transformer, VGG16, ResNet50), followed by temporal modeling with a transformer encoder and temporal convolution. This model was compared with conventional frame-based classification across 3 random train-test splits.

**Results:**

The dataset included 105 CLE sequences (3,173 images, 40 regions of interest). The sequence-based model achieved top-1 classification accuracies of 93% (vision transformer), 88% (VGG16), 74% (ResNet50), and 67% (Inception-ResNet-V2), outperforming corresponding frame-based models (78%, 74%, 55%, and 50%, respectively). Diagnostic performance was comparable to expert neuropathologist interpretation (87%). The model demonstrated robustness in artifact-affected sequences and improved interpretability by incorporating temporal progression.

**Conclusions:**

AI models that integrate both visual and temporal information from CLE digital imaging sequences can effectively classify brain tumor grade with accuracy comparable to that of expert neuropathologists, outperforming frame-based models. Such a system reduces interpretive subjectivity and holds promise as an intraoperative decision CLE support tool for nonenhancing brain tumor resection.

## 1 Introduction

The 2021 World Health Organization (WHO) classification of central nervous system (CNS) tumors has significantly reshaped the diagnosis and management of gliomas and other primary brain tumors, emphasizing molecular parameters alongside histopathological features (1, 2). This updated classification has stimulated advanced research in radiomics and multi-omics profiling to improve brain tumor subtyping and guide precision therapeutics (3, 4). This shift has important implications for nonenhancing gliomas, which occur in adult and pediatric populations (5, 6). Thirty to forty percent of gliomas that do not enhance on preoperative magnetic resonance imaging (MRI) harbor anaplastic regions or are completely malignant according to previously published studies (7, 8). Based on their imaging appearance, these tumors are often mistakenly interpreted preoperatively as “low-grade” gliomas and thus are not properly categorized until intraoperative tissue assessment is performed. Proper identification and biopsy of these more aggressive regions during surgery is crucial to avoid histological undergrading and guide appropriate adjuvant treatment.

Confocal laser endomicroscopy (CLE) is a US Food and Drug Administration–cleared, real-time intraoperative imaging modality that captures cellular resolution images of brain tissue using fluorescein sodium (FNa) as a contrast agent. During tumor resection, the surgeon-handheld CLE probe comes into contact with the tissue surface, generating approximately 1 image every 1.3 s. After being given intravenously (in usual circumstances and according to current neurosurgical protocol, approximately 5–10 min before imaging), FNa leaks through the abnormal tumor blood brain barrier. FNa remains in the extracellular space, and its fluorescence lights up the background of the images. The cells are represented as dark round silhouettes of various sizes, morphology, and density. A few minutes of intraoperative imaging results in sequences that often comprise dozens or even hundreds of images within a single examination session. Neuropathologists and neurosurgeons interpret these sequences of CLE images in real time to differentiate between lesional and healthy tissue based on various characteristics revealed by the images, such as cellular density, cellular heterogeneity, and histoarchitectural background.

At tumor margins, CLE has demonstrated excellent sensitivity for detecting tumor infiltration. Additionally, this tool has shown promise in assisting with the determination of tumor (i.e., glioma) grade during resections of nonenhancing brain tumors. However, the interrater reliability of CLE interpretation by neuropathologists who are well-trained in CLE imaging is significantly lower than that associated with conventional hematoxylin and eosin (H&E)–stained pathology images, especially at the tumor margins, suggesting an inherent uncertainty in CLE images that contributes to subjectivity in their interpretation (9). Efforts have therefore been made to enhance the interpretation of CLE images. Furthermore, nonenhancing tumors present unique challenges in CLE imaging because they have relatively intact blood-brain barriers that are less permeable to gadolinium contrast and FNa diffusion, resulting in darker images with pathognomonic features or histoarchitectural content that is difficult to identify (10).

With the FNa-based CLE system, about 50% of images show artifacts due to movement either by the surgeon or the brain itself, or by red blood cells which can be confused with small tumor cells. The number of images collected can become overwhelming, especially when attempting to select relevant, informative, and actionable images for the surgeon. Artificial intelligence (AI) models have been proposed to detect diagnostic frames, transform grayscale images into H&E-stained histology images, localize diagnostic features, and classify CLE images (11–15). One common downside of these methods is that the datasets consist of single independent CLE images, thereby ignoring the sequential information that human interpreters often incorporate when the images are sequenced. This difference creates a fundamental gap between human interpretive behavior and the current AI model design.

This feasibility study aimed to bridge that gap by developing and applying an AI system that models the sequential visual reasoning process used by expert human interpreters when interpreting CLE image sequences. By capturing temporal context and visual progression across frames from sequentially acquired CLE images, our proposed system seeks to facilitate more accurate, consistent, and clinically relevant intraoperative assessments of tumor grade in brain tumors that do not significantly enhance on preoperative MRI. Such a system could enhance surgical decision-making when using CLE to evaluate these diagnostically complex tumors.

## 2 Materials and methods

### 2.1 CLE image acquisition

The CLE images used in this study were collected during clinical studies conducted at Barrow Neurological Institute and approved by the Institutional Review Board of Human Research at St. Joseph's Hospital and Medical Center (16, 17). We used AI techniques to analyze CLE images acquired intraoperatively from patients who underwent brain tumor surgery at Barrow Neurological Institute, St. Joseph's Hospital and Medical Center, Phoenix, Arizona, between 2010 and 2023. All participants provided written informed consent prior to their participation.

Only CLE images from nonenhancing brain mass lesions identified on preoperative MRI, along with confirmed histopathology and assigned WHO CNS grade, were included in the analysis. Images were acquired using 1 of 2 CLE systems: a clinical system (CONVIVO, Carl Zeiss Meditec AG, Jena, Germany) or a preclinical system (Five1, Optiscan Imaging Ltd., Mulgrave, Australia). Both systems use similar technology developed by Optiscan Imaging Ltd. and generate comparable image quality, differing mainly in the user interface, image dimensions, and functional features. For all cases, FNa was administered intravenously at a dose of 5 mg/kg within minutes before CLE image acquisition. CLE images with H&E-stained histologic sections from the same imaging spot were collected for analysis.

### 2.2 CLE image interpretation by a neuropathologist

All CLE images were reviewed by a single neuropathologist (J.M.E.) with 15 years of experience and expertise in interpreting CLE images. Each CLE region of interest (ROI) was evaluated, and an overall high- or low-grade interpretation was assigned to the entire sequence. The neuropathologist remained blinded to the final pathological diagnosis during the evaluation. Atypical features (e.g., hypercellularity, cellular pleomorphism, necrosis, and microvascular proliferation) were documented and used as indicators of higher tumor grade and malignancy. In contrast, normal or mildly increased cellularity without evident cellular pleomorphism suggested lower-grade tumors. CLE interpretations were subsequently compared with the corresponding histopathological diagnosis and H&E-stained sections, which served as the ground truth for the neuropathologist's interpretation and the AI model's classification.

### 2.3 Data preprocessing for AI-based CLE image analysis

After manually excluding CLE image sequences entirely affected by blood or motion artifacts, the remaining images underwent standardized preprocessing, which consisted of 2 main steps: (1) normalizing image dimensions to the [0,1] range to ensure numerical stability, maintain dataset consistency, and prevent gradient explosion during the training process; and (2) downsizing the image to meet the input requirements of the vision backbone architectures.

### 2.4 Neural network design for the sequence-based approach

**Figure 1:**
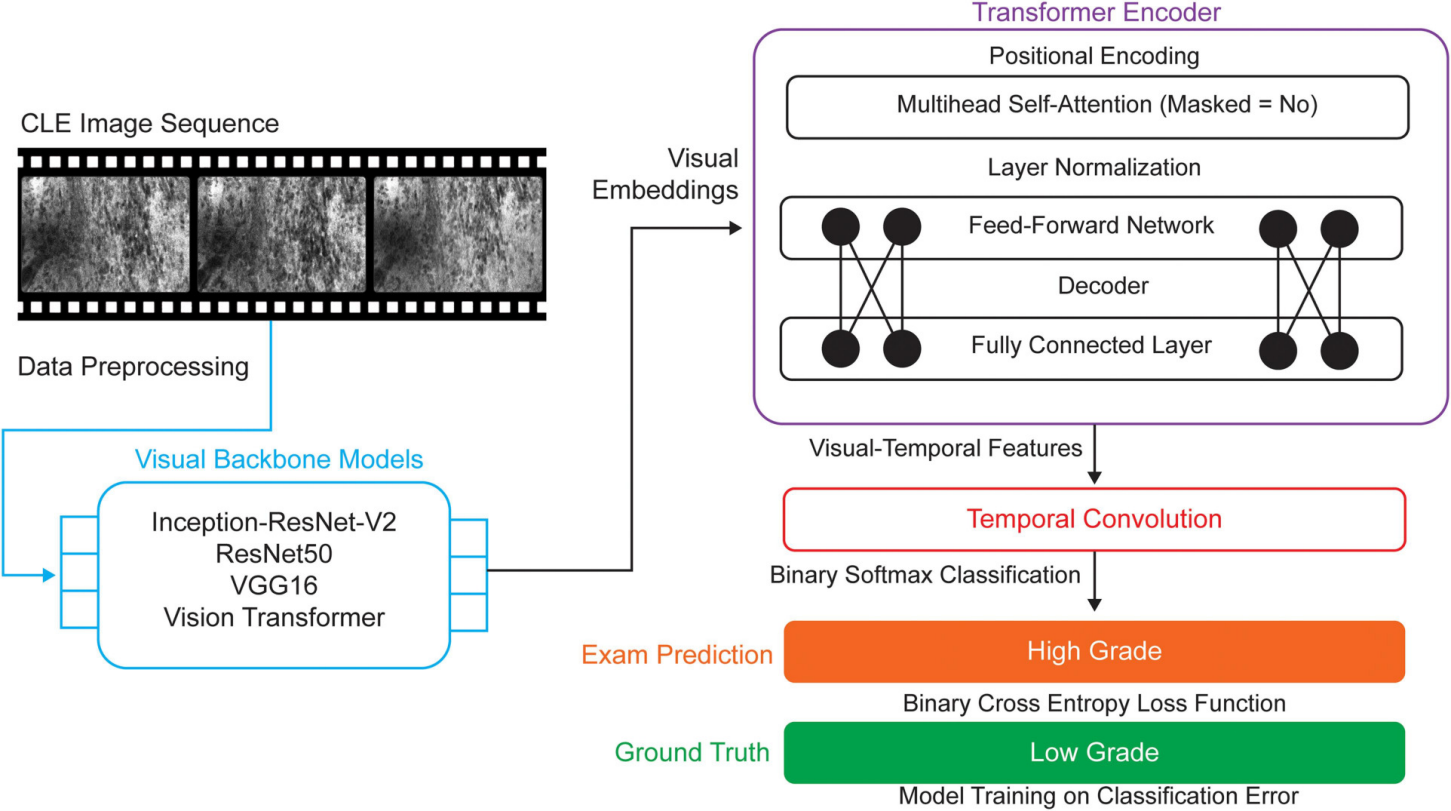
The architecture of the newly proposed sequence-based classification model is designed to capture both spatial and temporal information from confocal laser endomicroscopy (CLE) image sequences. Following standardized preprocessing, each frame within a sequence is passed through 1 of 4 visual backbone models (Inception-ResNet-V2, ResNet50, VGG16, or vision transformer) to extract high-dimensional spatial feature embeddings. These visual embeddings are then input into a transformer encoder, which models temporal dependencies across the sequence, followed by a temporal convolutional layer that further aggregates sequential information and reduces dimensionality. The resulting visual-temporal representation is fed into a fully connected layer with a Softmax activation to produce a binary prediction label, which is compared against the ground truth for tumor grade classification. *Used with permission from Barrow Neurological Institute, Phoenix, Arizona*.

Two approaches were employed for the AI-based interpretation of CLE images: a conventional single-frame approach (14, 15, 18) and a newly proposed sequence-based approach. A new neural network architecture composed of multiple extraction components was developed for the sequence-based approach (Figure 1).

#### 2.4.1 Visual feature extraction

First, to extract spatial features from each CLE image, we employed neural network models that were pretrained on the ImageNet V2 dataset as visual backbone architectures (19, 20). Several well-established architecture models [Inception-ResNet-V2 (21), ResNet50 (22), VGG16 (23), and vision transformer (24)] have demonstrated strong performance in medical imaging and proven effectiveness in image representation learning. To comprehensively evaluate their performance on our dataset, all 4 architecture models were integrated into our model in parallel. Each visual backbone architecture processes CLE images and encodes them into high-dimensional feature vectors. For a given sequence of CLE images, this results in a corresponding sequence of images that serves as input to the subsequent temporal modeling module.

#### 2.4.2 Temporal modeling

The extracted visual features are passed into a transformer encoder to model the temporal relationships and dependencies across the CLE image sequence (25). To maintain the temporal order of CLE frames, positional encoding is added to the input feature sequence, allowing the model to learn the relative and absolute positions of frames within the examination. This enables the transformer encoder to model the relative and absolute positions of frames, which is essential for capturing disease progression patterns in sequential CLE images.

#### 2.4.3 Temporal convolution and dimensional reduction

To further aggregate temporal information and reduce the sequence dimension, the output of the transformer encoder is passed through a 1-dimensional temporal convolutional layer (26). This layer applies filters across the temporal axis to capture local temporal patterns and combine information across neighboring frames. It also compresses the sequence into a single global temporal embedding vector that represents the entire CLE image sequence. Finally, the resulting latent visual-temporal feature is processed by a fully connected layer, followed by a Softmax classifier that outputs a binary label indicating tumor grade: high-grade tumor or low-grade tumor.

### 2.5 Model training and testing

We randomly divided the dataset of 105 CLE image sequences into a training set (78 sequences, 74.3%) and a testing set (27 sequences, 25.7%). The neuropathologist's interpretation of the matching H&E section as high- or low-grade tumor was used as the ground truth label. To ensure robustness and minimize bias from randomization, we conducted experiments on 3 different random splits. In the training stage, we used the cross-entropy loss function to compute classification loss by comparing the predicted high and low tumor grades with the ground truth label. Model parameters were optimized using the adaptive moment estimator optimizer, which facilitated efficient backpropagation and convergence. During testing, the model processed CLE image sequences following the same procedure as in the training stage. Performance was evaluated using top-1 accuracy, calculated on a per-examination basis and reported for each test set in addition to sensitivity, specificity, positive predictive value (PPV) and negative predictive value (NPV). All experiments were conducted using a desktop NVIDIA GeForce RTX 3090 graphics processing unit (GPU) (24 GB memory).

## 3 Results

### 3.1 Descriptive analysis

In total, 105 CLE image sequences containing 3,173 CLE images from 40 ROIs across 16 cases were included in the analysis (Table 1). These comprised 2 astrocytomas (12%), 10 oligodendrogliomas (63%), 1 glioblastoma (6%), 2 glioneuronal and neuronal tumors (12%), and 1 miscellaneous mass lesion (6%). Eight (50%) were pathology-confirmed low-grade (WHO CNS grade 1 and 2) lesions, and 8 (50%) were pathology-confirmed high-grade (WHO CNS grade 3 and 4) lesions.

**Table 1:** Tumor histology type and WHO grade of 16 nonenhancing brain tumors.

Histology type	WHO grade	No. of tumors
Astrocytoma	3	2
Oligodendroglioma	2	5
	3	5
Glioblastoma	4	1
Glioneuronal and neuronal tumors	1	2
Miscellaneous	1	1

WHO, World Health Organization.

### 3.2 Neuropathologist interpretation

**Figure 2:**
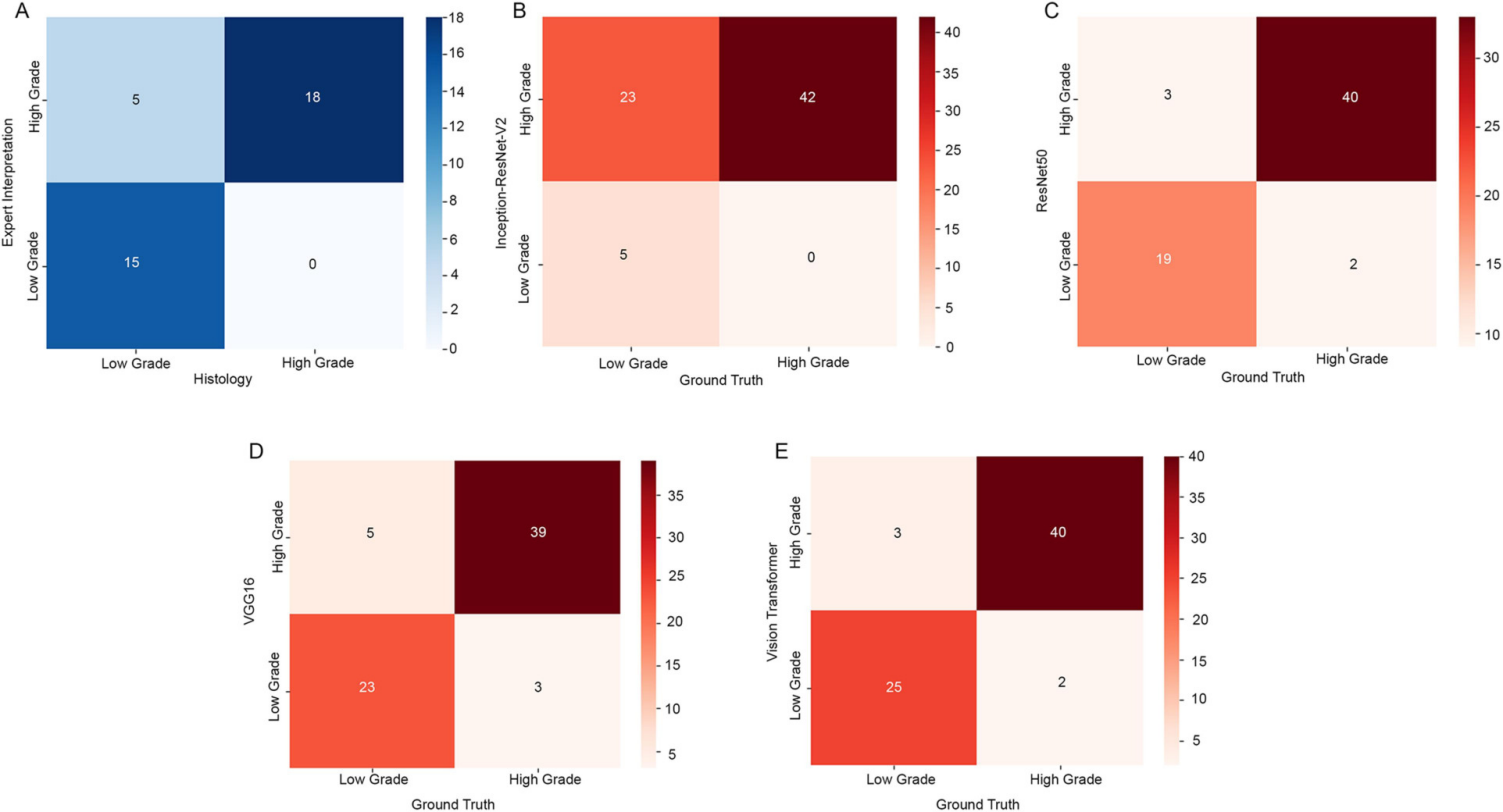
Confusion matrices comparing the interpretation by the human expert **(A)** and the artificial intelligence (AI) models’ classification **(B–E)** in differentiating high-grade and low-grade pathology. Each panel shows a confusion matrix in which the *x*-axis represents the ground truth (histologically confirmed tumor grade), and the *y*-axis represents the interpretation by the human expert or the predicted label by the AI model. Color intensity corresponds to the count in each cell, with darker colors indicating higher values. *Used with permission from Barrow Neurological Institute, Phoenix, Arizona*.

The neuropathologist successfully interpreted CLE images from 38 of 40 ROIs. Concurrent hypercellularity and pleomorphism were observed in 23 ROIs, with 18 classified as high-grade based on histology; 15 ROIs did not exhibit hypercellularity or pleomorphism, all corresponding to low-grade pathology (Figure 2A). The overall accuracy of the neuropathologist's interpretation was 87%.

### 3.3 AI model classification

To evaluate the effectiveness and clinical relevance of our proposed sequence-based CLE image classification framework, we conducted a thorough assessment focusing on 2 main aspects: (1) the influence of the 4 visual backbone models, and (2) the classification accuracy of our sequence-based method compared to traditional frame-based image classification.

#### 3.3.1 Visual model comparison

**Figure 3:**
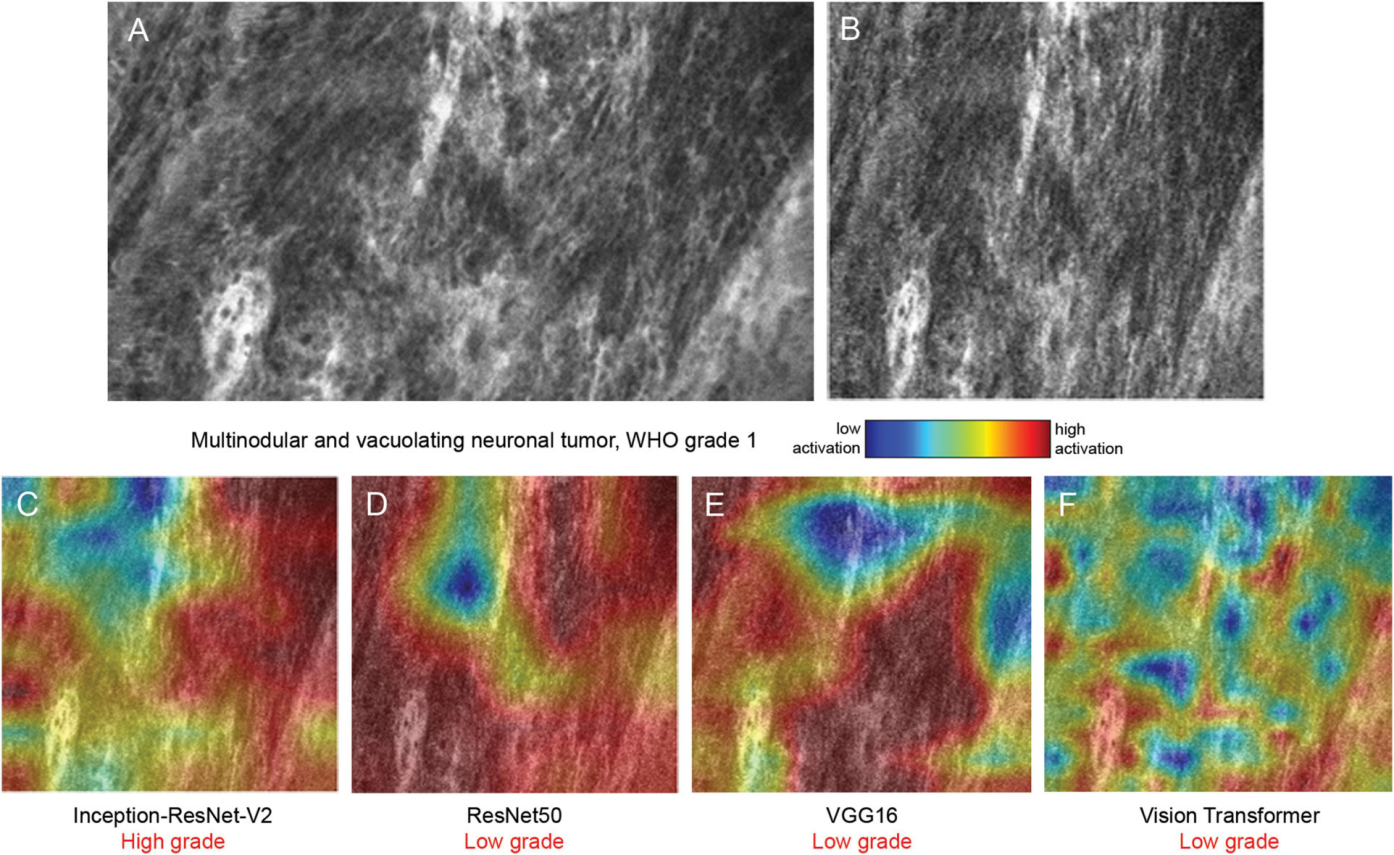
Class activation maps (CAMs) from 4 visual backbone models on a World Health Organization (WHO) grade 1 multinodular and vacuolating neuronal tumor sample. Representative confocal laser endomicroscopy (CLE) image in its original form **(A)** and after preprocessing **(B)**. CAMs generated by Inception-ResNet-V2 **(C)**, ResNet50 **(D)**, VGG16 **(E)**, and vision transformer **(F)** overlaid on the input image. Inception-ResNet-V2 misclassified the sample as high-grade, and ResNet50, VGG16, and vision transformer correctly classified it as low-grade. The CLE image sequence is shown in Supplementary Video S1. *Used with permission from Barrow Neurological Institute, Phoenix, Arizona*.

**Figure 4:**
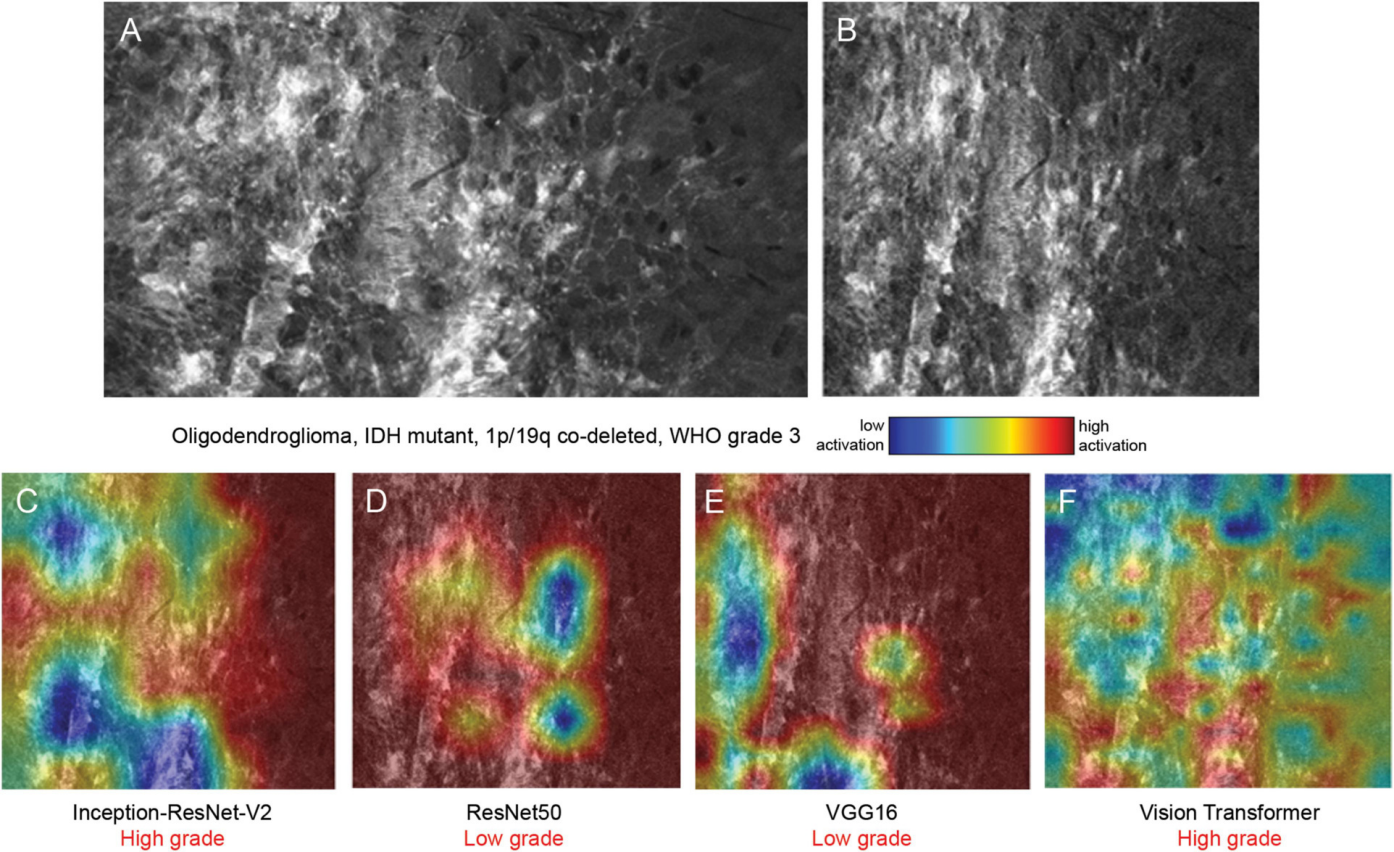
Class activation maps (CAMs) from 4 visual backbone models on a World Health Organization (WHO) grade 3 oligodendroglioma sample. Representative confocal laser endomicroscopy (CLE) image in its original form **(A)** and after preprocessing **(B)**. CAMs generated by Inception-ResNet-V2 **(C)**, ResNet50 **(D)**, VGG16 **(E)**, and vision transformer **(F)**, overlaid on the input image. ResNet50 and VGG16 misclassified the sample as low-grade, and Inception-ResNet-V2 and vision transformer correctly classified the sample as high-grade. The CLE image sequence is shown in Supplementary Video S2. *Used with permission from Barrow Neurological Institute, Phoenix, Arizona*.

**Figure 5:**
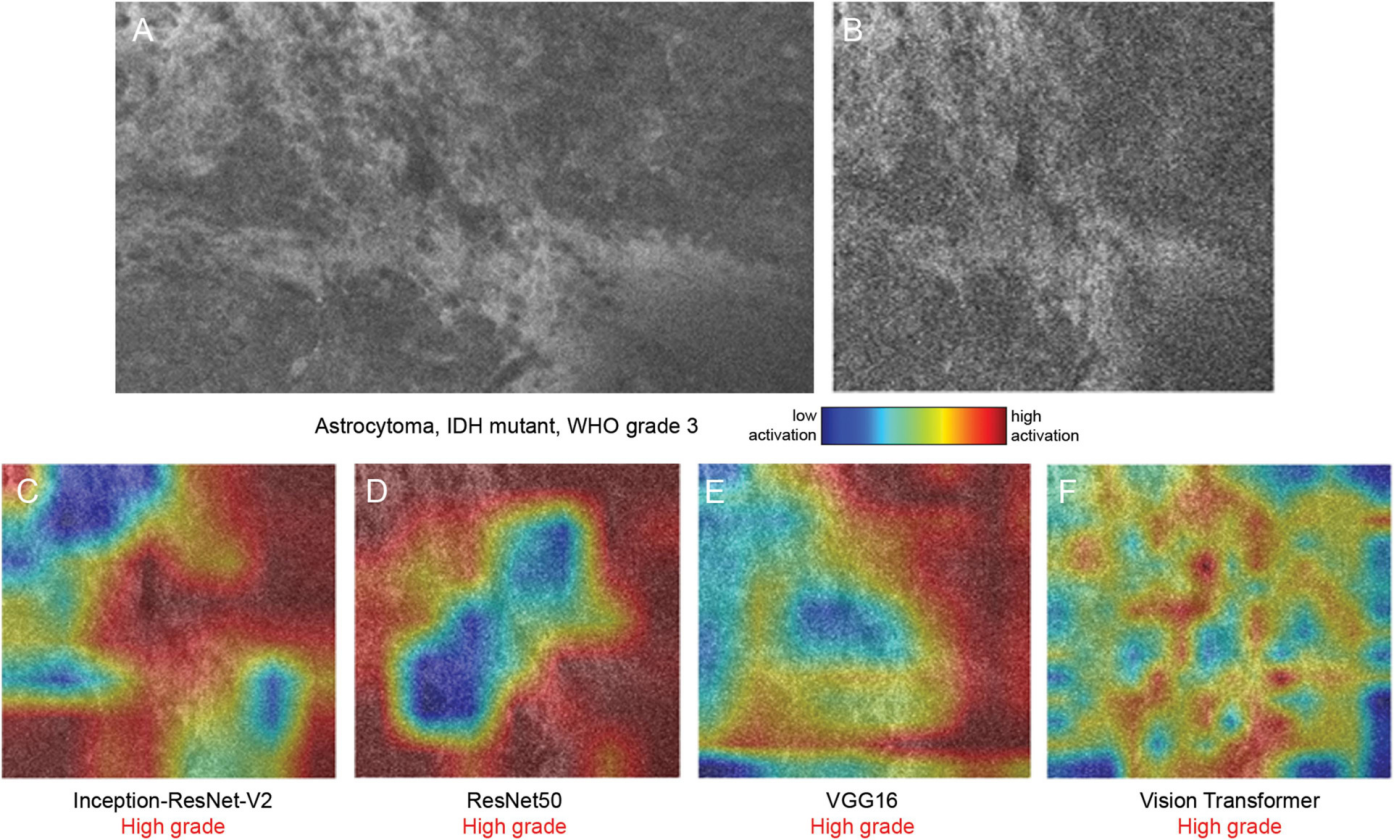
Class activation maps (CAMs) from 4 visual backbone models on a World Health Organization (WHO) grade 3 astrocytoma sample. Representative confocal laser endomicroscopy (CLE) image in its original form **(A)** and after preprocessing **(B)**. **(C–F)** CAMs generated by Inception-ResNet-V2 **(C)**, ResNet50 **(D)**, VGG16 **(E)**, and vision transformer **(F)** overlaid on the input image. Red to blue colormap indicates the degree of model activation, with warmer colors denoting stronger relevance to the predicted high-grade label. All 4 models correctly classified the sample as high-grade. The CLE image sequence is shown in Supplementary Video S3. *Used with permission from Barrow Neurological Institute, Phoenix, Arizona*.

Among the 4 visual backbone models, vision transformer exhibited the strongest performance in capturing complex spatial structures and achieved the highest overall classification accuracy, followed by VGG16, ResNet50, and Inception-ResNet-V2, respectively (Tables 2, 3). This indicated that transformer-based visual models, with their capacity to capture long-range spatial dependencies among image patches, might be particularly effective in analyzing CLE images in which subtle structural changes signal tumor grade. Confusion matrices illustrate the distinct tendency of the Inception-ResNet-V2 model to misclassify low-grade pathology as high-grade pathology (Figure 2B), whereas the classifications from the other visual models were more balanced (Figures 2C–E). Class activation maps (Figures 3–5) highlighted the areas of the input image that contributed most to specific class predictions.

**Table 2:** Top-1 accuracy of the frame-based classification model.

Split	Accuracy, %			
	Inception-ResNet-V2	ResNet50	VGG16	Vision transformer
1	49	53	77	82
2	53	53	70	75
3	49	60	74	76
Overall	50	55	74	78

**Table 3:** Top-1 accuracy of the sequence-based classification model.

Split	Accuracy, %			
	Inception-ResNet-V2	ResNet50	VGG16	Vision transformer
1	68	71	93	96
2	64	68	82	86
3	68	78	89	89
Overall	67	74	88	93

#### 3.3.2 Frame-based and sequence-based classification models

The classic frame-based classification model yielded overall top-1 accuracies of 78%, 74%, 55%, and 50% with vision transformer, VGG16, ResNet50, and Inception-ResNet-V2, respectively, as the visual backbone (Table 2). In comparison, the sequence-based model produced tumor grade classification with significantly higher top-1 accuracies (vision transformer, 90%; VGG16, 88%; ResNet50, 73%; Inception-ResNet-V2, 67%; Table 3). Furthermore, with vision transformer and VGG16, the performance of the sequence-based model was comparable to that of the human neuropathologist's interpretation (87%) with excellent sensitivity, specificity, PPV, and NPV (Table 4).

**Table 4:** Sensitivity, specificity, positive predictive value, and negative predictive value of the sequence-based model using vision transformer and VGG16 as the visual backbone.

	Vision transformer, %	VGG16, %
Sensitivity	92	92
Specificity	88	82
PPV	92	88
NPV	88	87

PPV, positive predictive value; NPV, negative predictive value.

## 4 Discussion

Since the introduction of CLE imaging technology, efforts have been made to apply machine learning and deep learning methods to assist in interpreting CLE images (11–15). However, earlier studies focused on the analysis of individual images. To our knowledge, this is the first study to process sequentially acquired images to extract temporal information. Our AI model performed well in predicting the tumor grade of nonenhancing brain tumors based on CLE imaging. It demonstrated promise in intraoperative diagnosis and guidance of surgical maneuvers related to the tumor, including identifying tissue regions for further imaging and biopsy.

### 4.1 Models for image analysis and classification

The 4 visual backbone models process images in different ways, as evidenced by the varying high-activation regions (Figures 3–5). For Inception-ResNet-V2, ResNet50, and VGG16, the neural networks appeared to be activated to different degrees by the areas of high cellular density (Figures 3C–E, 4C–E, 5C–E), whereas the specific activation patterns did not always align with the regions that human experts focus on. Therefore, such AI model applications offer valuable dimensions to image analysis that can identify, concentrate on, and interpret features that might be overlooked by human interpretation or involve subtleties with significant implications.

VGG16 is a classic deep learning model composed of many simple layers that use small convolutional filters to extract detailed visual features progressively. Its straightforward and uniform architecture allows the model to focus on local textures and visually distinct patterns, which is advantageous for analyzing CLE images in which dense cellular regions are often key indicators. In our study, VGG16 achieved an accuracy of 88% in the sequence-based classification task, closely following vision transformer. Although its reliance on localized features can be a limitation in more subtle or heterogeneous cases, the activation maps reveal strong and focused attention on hypercellular areas, which aligns with the model's tendency to respond to clear localized signals.

ResNet50 introduces shortcut or “residual” connections that allow the model to preserve information across layers, enabling it to learn more abstract patterns. Despite this innovative design, ResNet50 achieved a relatively low accuracy (73%) in our study. Although it excels at detecting obvious features, it is less effective at integrating global context, which is crucial in interpreting CLE images with diffuse or ambiguous findings. In activation maps, ResNet50's narrower focus was associated with misclassification errors, suggesting that it missed more subtle contextual cues, corresponding to prominent histoarchitectural features, in the overall frame (Figures 3D, 4D, 5D).

Inception-ResNet-V2 combines 2 advanced design concepts. The Inception module allows the model to analyze features at multiple scales simultaneously, and the residual connections help preserve information as it passes through many layers. This architecture can be overly sensitive to complex textures and subtle variations. The activation maps demonstrated excessive attention to what appears to resemble hypercellularity, whereas other parts of the image that showed low-grade features were ignored, indicating that the model tended to interpret benign structural complexity as malignancy (Figures 3C, 4C, 5C). This led to the obvious tendency to overclassify low-grade pathology as high-grade pathology, indicated by the confusion matrix (Figure 2B).

Vision transformer divides an image into small, fixed-size patches and converts each patch into a token composed of numbers. It then processes these tokens using self-attention mechanisms to learn how they are related to each other and build a global understanding of the image, without relying on traditional convolutional operations. Vision transformer produces more dispersed activation patterns due to its patch-based processing and lack of spatial inductive bias, making the high-activation areas less intuitive to human visual inspection and less evident in terms of histoarchitectural characteristics in the image (Figures 3F, 4F, 5F).

In a multinodular and vacuolating neuronal tumor, regions of hypercellularity were observed in the image, but there was no significant cellular atypia or heterogeneity. ResNet50, VGG16, and vision transformer accurately classified this sample as a low-grade tumor (Figures 3D–F, Supplementary Video S1), whereas Inception-ResNet-V2 appeared to focus on the hypercellularity and mistakenly classified the tumor as a high-grade pathology (Figure 3C). In another WHO grade 3 oligodendroglioma sample, both ResNet50 and VGG16 were activated by the hypercellular regions and incorrectly classified the tumor as low-grade (Figures 4D,E, Supplementary Video S2), whereas the activation of Inception-ResNet-V2 and vision transformer resulted in the correct classification (Figures 4C,F). All 4 visual models correctly classified the sample from a WHO grade 3 astrocytoma as high-grade, although the activation pattern varied significantly (Figure 5, Supplementary Video S3).

### 4.2 Extraction, classification, and reasoning of CLE image features

For our experiments, before preprocessing, CLE image sequences that were completely affected by red blood cell or motion artifacts were manually removed. We did not use convolutional neural network-based diagnostic frame detection model (27) to filter single artifactual images to avoid disrupting the entirety of the image sequences.

To extract temporal features from the CLE image sequences, we selected the transformer encoder. It is equipped with 2 multihead self-attention layers that enable it to capture long-range temporal correlations and the sequential progression of CLE image content. Recurrent neural network–based models, such as long short-term memory (LSTM) networks (28), are theoretically suitable for this task. However, these models operate in an autoregressive manner, predicting each output step based on the previous one, which can lead to recurrent error propagation over long sequences. In contrast, the transformer encoder allows for at-once inference across the entire sequence, mitigating this issue through the use of self-attention encoding in parallel.

We compared our sequence-based classification model against a conventional frame-based classification model as the baseline. Although simple, the frame-based model overlooks temporal progression and contextual continuity, often resulting in erratic or inconsistent predictions due to frame-level noise or artifacts. In contrast, our sequence-based classification model mimics how a human expert evaluates the entire CLE examination, leveraging temporal modeling with a transformer encoder and local pattern extraction embedded in temporal convolutions to create a robust, temporally coherent representation of the full examination. This led to more stable and clinically aligned predictions, especially in borderline cases or instances with consecutive images affected by blood or motion artifacts.

Importantly, our method mimics how human experts interpret CLE imaging in a real-world setting. Human experts not only assess single frames in isolation but also gather information from sequential frames. In the first clinical feasibility study with the CLE system, it was apparent that our CLE-trained neuropathologist preferred examining whole sequences to uncover histoarchitectural features that individual static images do not reveal (29). This distinction is crucial when differentiating between blood contamination and densely packed tumor cells. Small, uniformly sized, disc-shaped cells that flow across the field of view in consecutive frames are more likely to be red blood cells entering the CLE imaging site and can thus be differentiated from relatively stable, motionless tumor cells. Additionally, the human brain's ability to parse information from unaffected areas when examining a series of images partially affected by motion artifacts allows for better utilization of these otherwise unusable images.

By explicitly modeling CLE as a temporal sequence and predicting at the examination level, our system emulates these human reasoning processes. This alignment between model behavior and clinical workflow enhances both the interpretability and trustworthiness of AI-assisted diagnostics, making it a better candidate for real-world intraoperative support. In turn, all visual backbone models benefited from being embedded in our sequence-aware architecture, showing clear improvements over their standalone, frame-based counterparts.

### 4.3 Integration of AI-based CLE image analysis into current intraoperative workflow

There are several features that greatly enhance the integration of CLE imaging into current intraoperative neurosurgical workflow. Martirosyan et al. previously reported successful co-registration of the CLE probe with the neuronavigation system to localize the precise CLE imaging site (17). Recently, Muscas et al. demonstrated the feasibility of connecting the CLE system (Zeiss CONVIVO), the neuronavigation system (Medtronic StealthStation S8), and the surgical microscope (Zeiss Kinevo 900) to enabled picture-in-picture display of CLE images in the surgical microscope visual field. This configuration reduced CLE imaging time and increased the proportion of usable CLE images by allowing the surgeon to assess image quality without diverting their gaze from the operative field (30). In addition, a built-in telepathology software platform allows for real-time transfer of CLE images from the operating room to the neuropathologist's device, along with voice communication between the neurosurgeon and neuropathologist (31).

Coupling our sequence-based AI model with an integrated surgical visualization ecosystem could provide neurosurgeons and neuropathologists with additional diagnostic information. This model can potentially be generalized to other CLE and intraoperative imaging systems that generate sequential images. Importantly, our approach is not limited by computing power. We used a consumer-grade GPU for model training and testing. For the 4 visual backbones, average inference times per sequence were as follows: Inception-ResNet-V2, 0.17 s; ResNet50, 0.11 s; VGG16, 0.19 s; and vision transformer, 0.33 s. With cloud-based deployment, our model can be pretrained and executed remotely on professional AI-optimized GPUs to provide near real-time feedback after image acquisition, enabling seamless integration with intraoperative CLE workflow.

Combined with *in vivo* CLE imaging, this tool would allow immediate intraoperative sampling of multiple spots within a nonenhancing tumor. Regions suspicious for higher tumor grade can be identified and biopsy performed to further confirm tumor grade and potentially avoid histological undergrading. Integration of such an AI-assisted system into the intraoperative workflow may ultimately enhance surgical precision and improve diagnostic yield while streamlining decision-making and improving workflow efficiency during brain tumor surgeries. However, prior to being implemented in clinical practice, our AI model needs to be refined. A more comprehensive dataset collected from multiple institutions and annotated uniformly should be used to further train and validate the model. A more balanced distribution of tumor histology type and WHO grade would be highly desirable. Prospective clinical studies should be considered to determine the reliability, clinical benefit, and workflow impact of the model.

### 4.4 Limitations

The relatively small sample size and single-institution nature limited this study. However, the point of this study was a proof of principle, procedure, and exploration of appropriate and applicable AI image assessment programs to engage with the modes of CLE image acquisition and interpretation. All CLE images were acquired at a single neurosurgical center using standardized imaging protocols and interpreted by a single neuropathologist, which may not fully represent the variability of intraoperative imaging conditions, tumor pathologies, and inter-rater variability across other institutions. To mitigate potential bias and reduce randomization errors from assigning CLE image sequences to training and testing sets, we repeated all experiments across 3 different random splits and reported the average top-1 accuracy. This approach aimed to ensure a more robust and reliable performance evaluation despite the dataset size constraint. The results of this study will inform sample size calculations for future, larger-scale experiments aimed at validating the conclusions of this preliminary work. Furthermore, more than half of the cases in our dataset were oligodendrogliomas, whereas astrocytomas were significantly underrepresented. This may limit the generalizability of our results; nevertheless, we consider this feasibility study to be an important first step. Future expansion of the dataset with a more balanced representation of tumor types will likely enhance the robustness and performance of our model.

## 5 Conclusions

This study presents a novel image sequence–based AI framework that significantly enhances the interpretation of CLE image sequences for intraoperative assessment of surgically and diagnostically complex nonenhancing brain tumors. By incorporating temporal context through a transformer encoder and integrating advanced visual backbone models, the proposed system outperforms conventional frame-based approaches, achieving accuracy comparable to that of an expert neuropathologist. This combined visual-temporal approach not only improves classification consistency and robustness, particularly in sequences affected by artifacts, but also aligns closely with the real-world interpretations of human experts. When hundreds to thousands of CLE images are acquired intraoperatively in real time, such AI systems may rapidly interpret images, especially those of brain tumors (i.e., gliomas) that exhibit high heterogeneity or possess complex imaging features. These findings highlight the potential of merging sequence-based deep learning models with CLE to support clinical decision-making during nonenhancing tumor resections, thereby reducing subjectivity in CLE interpretation and streamlining intraoperative surgical decisions.

## Data Availability

The original contributions presented in the study are included in the article/Supplementary Material, and further inquiries can be directed to the corresponding author.

## Supplementary material

Supplementary Video S1 A confocal laser endomicroscopy image sequence from a case of WHO grade 1 multinodular and vacuolating neuronal tumor. *Used with permission from Barrow Neurological Institute, Phoenix, Arizona*.

Supplementary Video S2 A confocal laser endomicroscopy image sequence from a case of WHO grade 3 oligodendroglioma. *Used with permission from Barrow Neurological Institute, Phoenix, Arizona*.

Supplementary Video S3 A confocal laser endomicroscopy image sequence from a case of WHO grade 3 astrocytoma. *Used with permission from Barrow Neurological Institute, Phoenix, Arizona*.
